# When donor funding ends: reflections from Jordan’s Health Care Accreditation Council on sustainable health system strengthening

**DOI:** 10.1093/intqhc/mzag033

**Published:** 2026-03-15

**Authors:** Salma Jaouni, Peter Lachman, Samar Hassan

**Affiliations:** Health Care Accreditation Council, Amman, Jordan; Patient Safety Movement Foundation, Directror of Patient Safety Fellowship, Irvine, CA, US; Health Care Accreditation Council, Amman, Jordan

Efforts to strengthen health systems in low- and middle-income countries (LMICs) often rely on donor-funded programs to accelerate reforms, build capacity, and introduce new models of care. While such investments can yield substantial progress during the funding period, sustainability frequently falters once external support ends. Similar challenges have emerged globally: in Ghana, district-level quality improvement teams weakened following the conclusion of donor-funded programs; in Kenya, accreditation reforms stalled after external technical advisors withdrew; and in Pakistan, provincial quality improvement frameworks failed to be fully embedded within public financing mechanisms [1, 2].

Against this global backdrop, the abrupt withdrawal of support from the United States Agency for International Development (USAID) in 2024 from three initiatives led by the Health Care Accreditation Council (HCAC) in Jordan offers a compelling case for reflection within the quality improvement community. Jordan’s experience is particularly instructive because it represents a relatively mature quality system that had transitioned toward national ownership, rather than a newly established or pilot stage intervention.

This paper does not recount the familiar donor-dependency narrative. Instead, it asks a more fundamental question: Why do health system strengthening initiatives struggle to endure beyond donor support, and how can sustainability be intentionally designed rather than assumed?

## Challenges to sustainability

Donor-funded initiatives typically pursue rapid improvements with ambitious deliverables tied to short-term project cycles [3]. The theory of change assumes that donor inputs lead to capacity building, strengthening systems, and eventual national ownership [4]. In practice, however, this transition is rarely automatic. Many programs successfully pilot new practices but fail to embed them within policy, financing, and institutional norms [5].

Key barriers persist across contexts. First, it may not align with national agendas, limiting political ownership. Second, core functions, such as data systems, training mechanisms, and accreditation programs, often lack domestic financing pathways [6]. Third, capacity transfer is sometimes incomplete, with reliance on external expertise persisting beyond project closure. Consequently, reforms remain vulnerable to disruption, particularly in settings with competing priorities or fiscal constraints. The problem is not a lack of impact during implementation but rather a systemic gap in planning for sustainability.

## The Jordan case

Over the past two decades, Jordan has been regarded as a regional model for health sector reform and quality improvement [7, 8]. Beginning in the early 2000s, development partners invested in quality assurance, accreditation, and primary healthcare reforms, laying the foundations for institutionalized quality across the health system. A major outcome of this period was the establishment of a national healthcare accreditation system led by the HCAC, which helped standardize quality practices, build institutional capacity, and promote a culture of quality in both public and private facilities.

As these reforms matured, the HCAC transitioned from a donor-supported initiative into an autonomous, nationally recognized body. Strong governance, technical rigor, and increasing national ownership enabled the adoption of self-sustaining strategies and formal integration within national health system structures, demonstrating the potential for donor-funded quality initiatives to evolve into durable institutions [9].

In 2025, this trajectory was disrupted by the unplanned cessation of support from the USAID, affecting three major initiatives led by the HCAC: the Health Services Quality Accelerator (HSQA), implemented with University Research Co.; the Community Health and Nutrition (CHN) initiative, implemented with Family Health International (FHI) 360; and the Local Health System Sustainability Project (LHSS), implemented with Abt Associates. The abrupt halt affected workforce development, national quality momentum, and long-standing partnerships.

While the HCAC remains operational and nationally anchored, this experience exposed ongoing vulnerabilities in the financing and continuity of quality-related functions. Jordan’s case reflects a broader pattern in many LMICs, where quality ­improvement systems remain partially dependent on external funding.

## Revealing structural fragilities

The premature termination of donor funding highlighted several vulnerabilities. Overreliance on external technical assistance left critical functions without local support. Domestic funding streams were insufficient to sustain quality improvement (QI) independently. More broadly, quality improvement tended to be treated as an ancillary aspect of development aid rather than an established national health priority [10].

## Lessons for sustainable health system strengthening

**Health system reforms endure when national institutions perceive them as their own.** Ownership is strengthened when local leadership drives decisions, codesigns interventions, and takes accountability, while external partners act as enablers aligned with national priorities.

**Reforms are more durable when embedded within national structures rather than delivered through parallel projects.** In Jordan, accreditation systems stabilized once institutionalized with legal and organizational backing.

**Sustainability is vulnerable without a clear transition plan.** Early planning for phased financial handover, diversified funding sources, and public financing mechanisms reduces dependency and protects essential functions.

**Resilience requires investing in local capacity and retaining it.** Strengthening expertise, institutional memory, and learning systems is more effective than relying on temporary external support. Retention strategies and clear career pathways ensure continuity.

**Sustainable reform relies on robust data and learning systems.** Monitoring mechanisms and performance platforms support feedback, adaptation, and continuous improvement beyond donor timelines.

## Re-imagining donor support for lasting impact

Experiences from Jordan illustrate broader challenges in sustaining donor-funded health system reforms. Early investments in governance, technical capacity, and embedding reforms within national structures can support resilience, yet abrupt funding withdrawals often expose vulnerabilities in continuity, workforce development, and long-term partnerships.

These experiences suggest that sustainable change requires deliberate attention to system integration, flexible support models, and domestic resource commitment. Overall, the durability of health system strengthening initiatives depends less on short-term gains and more on intentional planning, institutionalization, and long-term system thinking.

## Conclusion

Sustaining health system strengthening initiatives depends on local leadership, embedding reforms within national structures, and investing in capacity and governance. Donor funding can accelerate progress, but lasting impact relies on institutionalization and alignment with national priorities. Experiences from Jordan and similar contexts show that reforms are vulnerable without integration into broader health strategies.

## Conflicts of interest

None declared.

## Data Availability

The data underlying this article will be shared on reasonable request to the corresponding author.
